# Emotion Processing in Late Adulthood: The Effect of Emotional Valence and Face Age on Behavior and Scanning Patterns

**DOI:** 10.3390/bs15030302

**Published:** 2025-03-04

**Authors:** Bozana Meinhardt-Injac, Nicole Altvater-Mackensen, Alexandra Mohs, Jean-Christophe Goulet-Pelletier, Isabelle Boutet

**Affiliations:** 1 Department of Psychology, Catholic University of Applied Sciences Berlin (KHSB), 10318 Berlin, Germany; 2 School of Humanities, University of Mannheim, 68161 Mannheim, Germany; altvater-mackensen@uni-mannheim.de (N.A.-M.); amohs@students.uni-mainz.de (A.M.); 3 School of Psychology, University Ottawa, Ottawa, ON K1N 6N5, Canada; jgoul014@uottawa.ca (J.-C.G.-P.); isabelle.boutet@uottawa.ca (I.B.)

**Keywords:** aging, emotion recognition, positivity effect, own-age bias, eye tracking, valence and arousal

## Abstract

Age-related differences in emotion recognition are well-documented in older adults aged 65 and above, with stimulus valence and the age of the model being key influencing factors. This study examined these variables across three experiments using a novel set of images depicting younger and older models expressing positive and negative emotions (e.g., happy vs. sad; interested vs. bored). Experiment 1 focused on valence-arousal dimensions, Experiment 2 on emotion recognition accuracy, and Experiment 3 on visual fixation patterns. Age-related differences were found in emotion recognition. No significant age-related differences in gaze behavior were found; both age groups looked more at the eye region. The positivity effect—older adults’ tendency to prioritize positive over negative information—did not consistently manifest in recognition performance or scanning patterns. However, older adults evaluated positive emotions differently than negative emotions, rating negative facial expressions as less negative and positive emotions as more arousing compared to younger adults. Finally, emotions portrayed by younger models were rated as more intense and more positive than those portrayed by older adults by both older and younger adults. We conclude that the positivity effect and own-age bias may be more complex and nuanced than previously thought.

## 1. Introduction

Emotion recognition, a key component of social cognition, refers to an individual’s ability to accurately interpret and identify the emotions of others. Emotion recognition plays a critical role in facilitating successful social interactions (Erickson & Schulkin, 2003; Jack & Schyns, 2015). This ability is not static; rather, it evolves over the adult lifespan, with challenges emerging in healthy older adults aged 65 and above (Gourlay et al., 2022). Numerous studies have documented the difficulties older adults face when decoding the facial expressions of emotions (Ebner et al., 2024; Gonçalves et al., 2018; Hayes et al., 2020; Ruffman et al., 2008). The present study explores the effects of stimulus valence and face age on the interpretation, recognition, and visual scanning of facial expressions of emotions in younger and older adults.

### 1.1. Stimulus Valence

The majority of research investigating aging in emotion recognition has adopted the basic emotion model proposed by Ekman and Friesen (1978). This model posits that six basic emotions—happiness, anger, sadness, fear, disgust, and surprise—are inherently discrete, culturally universal, and physiologically distinct. These emotions are often used as a foundation for understanding how individuals interpret emotional cues from facial expressions, both in terms of theoretical constructs and methods. As such, research on age-related differences in emotion processing has focused on the recognition of the six basic emotions. Several studies on aging and emotion recognition have reported an interaction between participant age and stimulus valence. Specifically, there is an age-related decline in the accuracy of recognizing anger, fear, and sadness, while recognition of happiness tends to remain relatively intact (Gonçalves et al., 2018; Hayes et al., 2020; Ruffman et al., 2008). This phenomenon, often referred to as the “positivity effect”, can be interpreted through the lens of socioemotional selectivity theory (SST). According to SST, older adults perceive their future as limited in time, which motivates them to prioritize emotionally positive experiences (Carstensen et al., 1999; Charles & Carstensen, 2010). Consequently, older adults tend to allocate their processing resources toward stimuli that elicit positive emotions, thereby diverting attention away from negative information. This shift in focus allows them to optimize personal growth and maintain emotional wellbeing (Labouvie-Vief et al., 2010). Within the context of this framework, the positivity effect in emotion recognition is thought to reflect a motivation to pay more attention to elements that promote positive affect.

It has been argued that the positivity effect is confounded by differences in task difficulty and age-related variations in scanning patterns (Hayes et al., 2020). First, because much of the research on emotion recognition has focused on the six basic emotions, there is an imbalance between positive and negative stimuli (Hayes et al., 2020; Isaacowitz et al., 2007). As a result, ceiling effects are common for the recognition of happiness, probably because there is only one positive response option (i.e., happy) compared to four negative options (i.e., angry, sad, afraid, disgust). This imbalance confuses any interpretation of the positivity effect. To address this issue, Cortes et al. (2021) included an equal number of positive and negative emotions. They found significant group differences for six out of nine positive emotions, and for eight out of nine negative emotions. This suggests that when more positive emotions than happiness are included in the set of emotional expressions, the positivity effect is greatly diminished (Cortes et al., 2021). Moreover, most research on aging and emotion processing relies on stimulus sets that depict trained actors posing to convey intense emotions (e.g., by activating specific facial action units; see review by (Hayes et al., 2020). As a result, the arousal levels of the emotional stimuli utilized are artificial and excessively high (Bänziger et al., 2012). Second, it has been suggested that age-related differences in scanning patterns contribute to the positivity effect. There is evidence that younger adults tend to fixate more on the eyes, while older adults tend to fixate more on the mouth (Firestone et al., 2007; Grainger & Henry, 2020; Sullivan et al., 2007). Note that positive emotions such as happiness are primarily signaled in the lower half of the face, while negative emotions like anger, fear, and sadness are more closely associated with the upper half (Calder et al., 2000; Eisenbarth & Alpers, 2011). Therefore, it may be that older adults have less difficulty recognizing happy faces because this emotion is signaled by areas of the face on which older adults tend to focus. To further complicate matters, results comparing younger and older adults are sometimes contradictory. For instance, some eye-tracking studies have found that older adults employ a more explorative scanning strategy with more fixations across facial features when interpreting emotions (Eisenbarth & Alpers, 2011; Firestone et al., 2007; Stam et al., 2022). However, the contrary has also been reported with younger adults demonstrating more variability in their gaze patterns and older adults exhibiting more consistent fixations on the mouth (Chaby et al., 2017). These inconsistent findings highlight the need for further exploration of age-related differences in scanning strategies during emotion processing.

### 1.2. Valence and Arousal as Dimensions of Emotion Processing

While the basic emotion model conceptualizes emotions as discrete, other models posit that emotions exist along continuous dimensions, with valence and arousal being fundamental dimensions (P. J. Lang et al., 1993). Valence (positive/negative) refers to the direction of behavioral activation associated with emotion, either toward (appetitive motivation, pleasant emotion) or away from (aversive motivation, unpleasant emotion) a stimulus. Arousal (high/low) is proposed to be orthogonal to valence and refers to the intensity of the emotional activation, ranging from excited to calm. Emotional arousal reflects the motivational value of emotions (A. Lang et al., 2007) and enhances attention (Talmi et al., 2008) and memory (Cahill et al., 1996; Clark-Foos & Marsh, 2008). Valence and arousal independently contribute to emotional processing, with arousal effects preceding valence effects in early attentional stages (Recio et al., 2014).

Because most research on age-related differences in emotion processing aligns with the basic emotions model, few studies have explored how older adults process facial expressions in terms of valence and arousal dimensions. For emotional stimuli more broadly, behavioral studies suggest that older adults rate positive emotional images as less arousing (Grühn & Scheibe, 2008). Moreover, older adults show greater amygdala activation in response to positively-valenced stimuli (Kehoe et al., 2013). For facial expressions, older adults tend to rate negatively-valenced and neutral faces less negatively than younger adults (Czerwon et al., 2011). These findings, though limited, highlight valence and arousal as important dimensions warranting further study to enhance our understanding of aging and emotion processing. 

### 1.3. Face Age

In the domain of recognition of facial identity, there is evidence that both younger and older adults tend to recognize faces of one’s own age group more accurately than faces of another age group (Denkinger & Kinn, 2018; He et al., 2011; Rhodes & Anastasi, 2012). This effect is referred to as the own-age bias. In identity recognition studies, the own-age bias is attributed to the categorization of own-age faces as belonging to an ingroup, and to increased frequency of exposure to ingroup members. There is evidence that individuals have more confidence in their emotion recognition abilities when looking at images of individuals considered to be part of their ingroup (Beaupré & Hess, 2006). This suggests that an own-age bias may also exist for emotion processing. Yet, few studies have explored this question, likely because most existing emotion recognition stimulus sets predominantly feature younger adult models (Bänziger et al., 2012; Isaacowitz & Stanley, 2011; Phillips & Slessor, 2011). Some studies suggest that the own-age bias also exists in emotion recognition (Ebner et al., 2011; Ebner & Johnson, 2009). Others suggest that emotions are easier to recognize in younger faces irrespective of the age of the observer (Ebner et al., 2011). Given that the own-age bias may influence performance on emotion recognition tasks, it is important to further explore the impact of face age on emotion processing.

### 1.4. The Present Study

To address these gaps in the literature, we developed and utilized a new stimulus set that portrays younger and older models naturally expressing three positive (happy, interested, and friendly) and three negative (sad, angry, and bored) facial expressions. We used this new stimulus set to address the following research questions: (1) How does aging influence the processing of emotional expressions?; (2) How does emotional valence (positive vs. negative) affect emotion processing in young and older adulthood when there is a balance between positive and negative stimuli?; and (3) Does face age (younger vs. older models) influence emotion processing? To provide a more comprehensive understanding of the impact of participant age, stimulus valence, and face age on emotion processing, we measured various aspects of this phenomenon across three separate experiments. In Experiment 1, we focused on valence and arousal interpretations, in Experiment 2 on emotion recognition, and in Experiment 3 on eye scanning patterns.

## 2. Materials and Methods

### 2.1. Experiment 1: Valence and Arousal

#### 2.1.1. Participants

Participants were recruited using the Prolific platform. Participants were at least 18 years of age and had no prior history of neurological disorders or traumatic brain injury. The participants were categorized according to their age: individuals between the ages of 18 and 35 formed the younger adults (YA) group, and individuals over 60 formed the older adults (OA) group. Only participants who indicated their preferred language for reading and writing as English were able to participate as the survey was only available in English. A total of 189 participants completed the survey. Data from 37 participants were removed as they indicated participant ethnicities other than Caucasian, and three participants were removed because they failed to correctly answer at least three of the five engagement check questions. A total of 20 participants were removed because their completion time was 2 SD faster or slower than the mean of the sample. Our final sample size consisted of 129 participants: 59 YAs (age range: 18 to 35 years; M = 28 years, SD = 4.0 years) and 70 OAs (age range: 60 to 79 years; M = 68 years, SD = 4.3—for more details see Table 1: Exp. 1).

#### 2.1.2. Facial Expression in Aging Test (FEAT)

**Figure 1:**
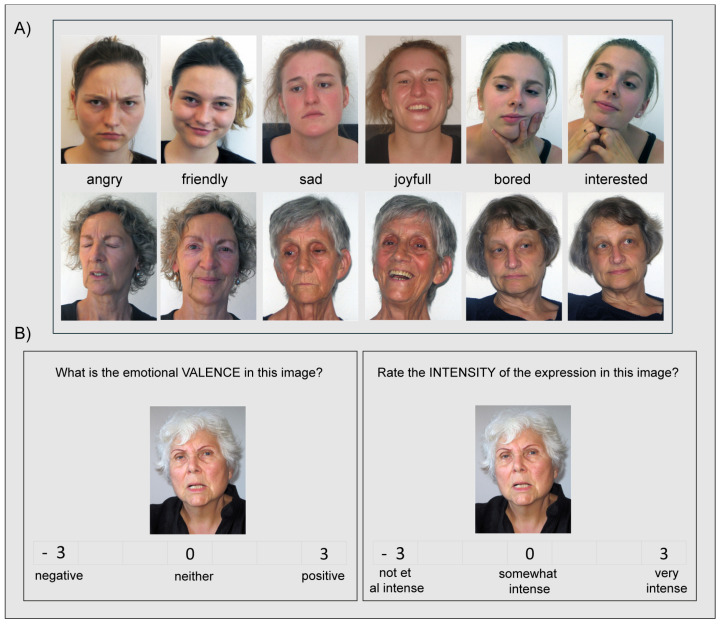
(**A**) Example of the face stimuli. (**B**) Trial example in Experiment 1.

Face models included 10 younger women (M = 23.5, age range: 19–26 years) and 10 older women (M = 72.3; age range: 62–81). All of the models were Caucasian and had an average appearance, without any obvious eye-catching features (i.e., jewelry or tattoos). They all wore black shirts and were photographed from the same distance (1.5 m). The background was always white, although the pictures were taken in different environments and under slightly varying lighting conditions (see Figure 1). The models were not trained actors posing to convey artificial emotions (e.g., by activating specific facial action units). Instead, participants were encouraged to use mental imagery and re-live personal events when experiencing the target emotion (Bänziger et al., 2012). For each emotion, a series of pictures were taken. Between different emotional states, there were short breaks in which the models were asked to think of something emotionally neutral. The next round of shooting started when the model felt ready to express the next emotion. On average 150 pictures were taken with each model (range: 120–200). Details about the test construction, and especially image selection, low-level features of the images, and distractor construction, are provided in Supplementary Material (see Section S1, Figure S1). In the end, the FEAT comprises 72 photos of 16 female models expressing six emotions: three positive emotions (happy, friendly, and interested) and three negative emotions (sad, angry, and bored)—see Figure 1). For each emotional expression, there were 12 photos posed for by six younger and six older models. This meant that not all six emotions in the final set were posed for by all 16 models. Moreover, as recommended for complex images, we calculated RMS contrast for each image (Peli, 1990). RMS contrast values are provided in Supplementary Material in Tables S1 and S2. For an overview of which emotional state was posed for by which model, please see Tables S1 and S2 in the Supplementary Material. The FEAT stimulus set is freely available: https://www.boutetscopelab.com/datasets (accessed on 24 February 2025).

The main aim of Experiment 1 was to measure how YAs and OAs process emotional expressions with regards to valence and arousal dimensions.

**Valence**: Participants were asked to rate the emotional valence of each image using a 7-point Likert-scale with negative and positive as the anchors (−3 to 3—see Figure 1B). For the data analysis, data were scored as follows: −3 = 1; −2 = 2; −1 = 3; neutral = 4; +1 = 5, +2 = 6, +3 = 7. This meant that emotions with scores >4 had positive valence, and emotions with scores <4 were negative in valence. Rating stimuli on valence helps to ensure that the intended emotional response aligns with the emotional category being studied (e.g., happiness, or sadness). It also allows for an assessment of whether YAs and OAs perceive the valence of stimuli similarly.

**Arousal**: Participants were asked to rate the intensity of the emotion expressed using a 7-point Likert-scale with not at all intense (−3) and very intense (+3) as the anchors (see Figure 1B). Again, for the data analysis, data were scored as follows: − 3 = 1; −2 = 2; −1 = 3; neutral = 4; +1 = 5, +2 = 6, +3 = 7. This meant that emotions with scores >4 had strong arousal, and emotions with scores <4 had low arousal. Rating stimuli on arousal helps distinguish between emotions that may have similar valence but differ in arousal levels (e.g., excitement vs. calmness). This differentiation is crucial for understanding how age might influence emotional processing.

### 2.2. Experiment 2: Age-Related Differences in Accuracy of Emotion Recognition (ER)

In Experiment 2, we investigated how emotional valence (positive vs. negative) and face age (younger model vs. older model) influenced emotion recognition accuracy among individuals in young and late adulthood. Emotion recognition was measured in a large sample of younger (18–39 years) and older adults (60–87 years). This allowed us to study age-related changes in emotion recognition.

#### 2.2.1. Participants

A total of 854 participants were recruited from a variety of sources, including through personal networks, social media forums, and website advertisements. Participants were also enrolled via a student pool, which included regular students as well as u3a students (i.e., Studium Generale for students aged 50 and over) at the Johannes Gutenberg University Mainz. The demographic characteristics of this sample are provided in Table 1: Exp. 2.

#### 2.2.2. Materials and Procedure

**Figure 2:**
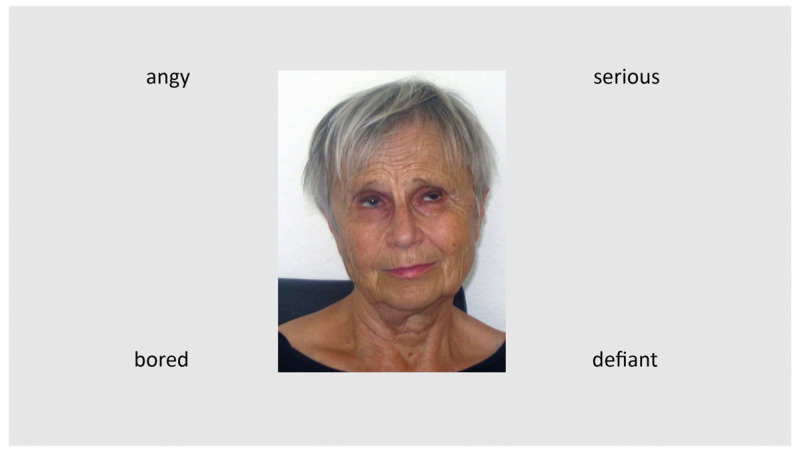
Trial example in Experiment 2: The target emotion is “bored”. The distractor words are “serious”, “defiant”, and “angry”. The presentation time was not limited. A complete list of target and distractor emotional words for each stimulus can be found in Table S1 (in German) and Table S2 (in English) in the Supplementary Material.

Data collection for Experiment 2 took place online (Wave 1 and Wave 2) and in the laboratory (Wave 3). Because the overall pattern of results was consistent across the three waves of data collection, data was pooled across the three waves. Descriptive and inferential statistics for the three data collection waves are provided in the Supplementary Material (see Sections S2 and S3, and Figure S2). Each wave of data collection was completed within the context of a larger research project where participants were tested on other measures (e.g., social network quality, facial identity recognition etc.) in addition to the emotion recognition test. Because these measures differed across studies, we only report data for the emotion recognition test in this article. For the emotion recognition test, one picture was chosen at random from the set of 72 for each trial. The picture was shown surrounded by four emotional words (see Figure 2). One word describes the target emotion, the others are distractors.

Participants were instructed to use the mouse to select the word that best describes the emotion shown. The stimulus was presented until the decision was made. For the whole sample, internal reliability was found to be acceptable (Cronbach’s α = 77; YA: α = 0.75, OA: α = 0.71) (Cicchetti, 1994; Cronbach, 1951). While reliability is rarely reported in the literature, our results are comparable to those available when it is reported. We refer the reader to Olderbak et al. (2021) for a review. More information on reliability can be found in Supplementary Materials (see Section S4).

### 2.3. Experiment 3: Age-Related Differences in Eye Gaze

In Experiment 3, we examined whether eye gaze scanning strategies for emotional expressions vary between younger and older adults. We also examined whether face age (younger vs. older models) influences these visual scanning patterns. We utilized the same stimulus set and procedure as in Experiment 2, except for minor procedural adaptations to accommodate eye-tracking methodology (see Section 2.3.2).

#### 2.3.1. Participants

The sample comprised 39 participants including both younger (age range: 18 to 34 years; M = 24.2 years, SD = 4.2 years) and older (age range: 60 to 75 years; M = 68.7 years, SD = 4.3 years) adults. Younger participants were recruited via the same student pool as Experiment 2 (i.e., we recruited regular students as well as u3a students (i.e., Studium Generale for students aged 50 and over) from Johannes Gutenberg University Mainz).

Of the initial 39 participants, 5 were excluded from the data analysis due to poor data quality, technical errors, or missing age information. The final sample included 17 participants from the younger and older groups, respectively. The demographic characteristics of the sample are provided in Table 1: Exp. 3.

#### 2.3.2. Materials and Procedure

The eye-tracking experiment was designed and conducted using OpenSesame (Mathôt et al., 2012). Participants were tested in a quiet testing room at the university. After participants were briefed about the study and provided their informed consent, they were seated 1.5 m in front of a screen (69 × 124 cm) on which stimuli were projected (24.5 × 19.5 cm). Each trial began with a white letter-number code displayed at the center of a black screen for 995 milliseconds, which directed the participants’ attention to the screen’s center. This was followed by the presentation of face stimuli against a white background for 1995 milliseconds (henceforth referred to as the scanning phase). Subsequently, emotional words appeared in the upper and lower left and right corners of the screen and remained visible until the trial concluded (henceforth, decision phase). One of these words served as the target, while the other three served as distractors. Participants verbally identified the word that best described the displayed emotion, prompting the experimenter to press a corresponding button to initiate the next trial. If participants did not respond within 11 s, the trial was automatically terminated, and the next trial began.

The experiment comprised six blocks, each containing 12 trials, resulting in a total of 72 trials per participant (i.e., each of the FEAT items was displayed once). The order of blocks and trials within each block was randomized. Each experimental block included six faces depicting positive emotions and six depicting negative emotions, with an equal distribution of younger and older adult models. The combination of faces and emotions within each block was counterbalanced.

Eye gaze patterns were recorded with Tobii Pro Glasses 1, employing a 5-point calibration. Eye movements were recorded at a rate of 60 Hz.

#### 2.3.3. Eye-Tracking Data Analysis

For the visualization and analysis of eye-tracking data, we utilized Tobii Pro Lab, Microsoft Excel and R Studio 4.2.2. Automatic mapping of eye gaze to the stimuli of interest (i.e., the presented faces) in Tobii Pro Lab was unsuccessful for a significant portion of the data. This was caused by failures to automatically identify the presented stimuli in the videos recorded from the participants’ view. For portions of the recordings in which a presented visual stimulus was clearly identifiable, but the automatic mapping failed, a trained coder locked the recording to the respective stimulus so that the gaze data could automatically be mapped and analyzed with respect to defined areas of interest (AOIs).

For the scanning phase in which only the face was visible, two non-overlapping areas of interest (AOIs) were defined: (1) the eyes (1554 × 838 pixel) and (2) the mouth (1538 × 857 pixel). The data, along with information on the AOIs, were exported from Tobii Pro Lab for all participants. A total of 92 trials were excluded due to missing data from skipped trials caused by experimenter errors or incomplete recordings of eye movements by the eye tracker (3% of data). The final dataset included 2356 trials with an average of M = 69.29 (SD = 6.19) trials per participant.

Eye tracking data was examined for age-related differences in fixations on the mouth and eyes in the scanning phase, i.e., before the emotional words appeared. Previous literature suggests that there are more fixations on the mouth compared to the eyes for older than for younger adults (Grainger & Henry, 2020). Additionally, positive emotions (i.e., happiness) are mainly expressed in the bottom half of the face whereas negative emotions are mainly expressed through the top half of the face (Calder et al., 2000). Based on this literature, we focused our analysis on the proportional duration of fixations on the mouth region, calculated as proportional mouth looking = total looking time to mouth AOI/(total looking time to mouth AOI + total looking time to eyes AOI). The proportional duration of participants’ fixations on the mouth was calculated on a trial-by-trial basis, thereby normalizing for differences in total looking time across trials and participants.

The following R Studio packages were utilized for data processing and analysis: reshape for data aggregation (Wickham, 2007), car for testing normal distribution and homoscedasticity assumptions (Fox & Weisberg, 2019), rstatix (Wickham & Sievert, 2016), for statistical modeling across aggregated data, and emmeans for post-hoc test calculations (Lenth et al., 2018) The Benjamini–Hochberg procedure was applied to control the false discovery rate due to multiple testing.

### 2.4. Ethics Statement

All data collected during the studies were handled in accordance with applicable data protection regulations. Ethical approval was obtained from the University of Ottawa, Canada, for Experiment 1 for the research project: “Investigating the perception of expressions of emotions” (Ethics File Number H-09-22-8395). Participants were informed about how their data would be used, stored, and shared, and were made aware of the nature of the task, ensuring transparency in the research process. Informed consent was obtained from all participants involved in Experiments 1–3. Participants were assured that their responses would remain confidential and that their participation was voluntary.

## 3. Results

### 3.1. Age Related Differences in Emotion Processing

#### 3.1.1. Experiment 1: Age Related Differences in Valence and Arousal

**Figure 3:**
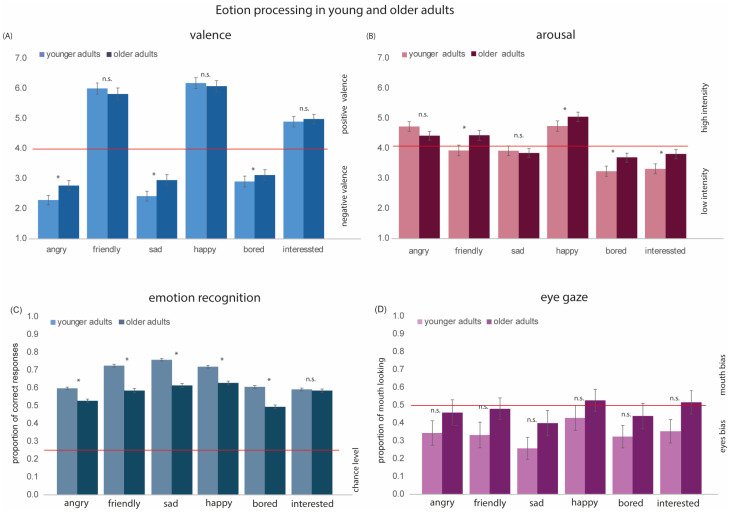
Results for each of the six emotions in younger and older adults. (**A**) Mean valence scores. Emotions with scores >4 (above the read line) had positive valence, and emotions with scores <4 (below the red line) had negative valence. (**B**) Mean arousal scores. Emotions with scores >4 (above the red line) had strong arousal, and emotions with scores <4 (below the red line) had low arousal. (**C**) Mean accuracy of emotion recognition. The red line represents the chance level. The proportion of correct responses for all emotions was above the chance level. (**D**) Proportion of mouth looking. For emotions with scores >5 (above the red line), there was a mouth bias, and for emotions with scores <5 (below the red line), there is an eyes bias. We see that both younger and older adults show a stronger looking preference toward the eyes in emotional stimuli. * denotes statistically significant effects; n.s. = not significant. Error bars indicate ± 1 standard error.

The valence and arousal ratings collected in Experiment 1 were analyzed using TIBCO^®^ Statistica Version 14.0.015. We conducted 2 × 6 repeated measures (rm) ANOVAs with Age Group (2; younger adults [YAs] vs. older adults [OAs]) as the between-group variable and Emotion (6; Angry, Friendly, Sad, Happy, Bored, and Interested) as the within-group variable. The main effects of Age Group [F(1, 127) = 10.96, *p* < 0.01, η^2^ = 0.07] and Emotion [F(5, 635) = 854.56, *p* < 0.001, η^2^ = 0.87] were significant. Overall, older adults rated faces more positively than younger adults. The interaction between Age Group and Emotion was also significant [F(5, 635) = 6.79, *p* < 0.001, η^2^ = 0.06]. Post-hoc *t*-tests indicated that older adults tended to rate negative emotions less negatively than younger adults (anger: t(127) = −2.77, *p* < 0.01; sadness: t(127) = −3.06, *p* < 0.005; boredom: t(127) = −2.01, *p* < 0.05). In contrast, older and younger adults rated positive emotions similarly (friendliness: *t*(127) = 1.53, *p* = 0.12; happiness: *t*(127) = 1.68, *p* = 0.09; interest: *t*(127) = 0.23, *p* = 0.81) (see Figure 3A).

For arousal, the main effects of Age Group [F(1, 127) = 7.63, *p* < 0.01, η^2^ = 0.05] and Emotion [F(5, 635) = 94.04, *p* < 0.001, η^2^ = 0.42] were significant. Overall, older adults rated faces as more arousing than younger adults. The interaction between Age Group and Emotion was also significant [*F*(5, 635) = 3.41, *p* < 0.01, η^2^ = 0.02]. Post-hoc *t*-tests indicated that older adults judged boredom [*t*(127) = −2.34, *p* < 0.05], friendliness [*t*(127) = −3.31, *p* < 0.01], happiness [*t*(127) = −2.33, *p* < 0.05], and interest [*t*(127) = 3.78, *p* < 0.01] as more arousing than younger adults (see Figure 3B). There were no significant differences in arousal ratings between younger and older adults for angry [*t*(127) = 0.53, *p* = 0.59], and sad [*t*(127) = −1.33, *p* = 0.18] emotional expressions.

#### 3.1.2. Age-Related Differences in Emotion Recognition

The proportion of correct responses for emotion recognition collected in Experiment 2 was analyzed using a 2 × 6 rm ANOVA with Age Group (2; younger adults [YAs] vs. older adults [OAs]) as the between-group variable and Emotion (6; Angry, Friendly, Sad, Happy, Bored, Interested) as the within-group variable. The main effect of Age Group [F(1, 852) = 128.61, *p* < 0.001, η^2^ = 0.13] was significant: younger adults displayed higher accuracy in emotion recognition than older adults. The main effect of Emotion [F(5, 4260) = 1166.41, *p* < 0.001, η^2^ = 0.12] and the interaction between Age Group and Emotion [F(5, 4260) = 39.85, *p* < 0.001, η^2^ = 0.04] were also significant. Post-hoc *t*-tests indicated age-related difference in the recognition of angry [*t*(852) = 5.8, *p* < 0.001], friendly [*t*(852) = 9.81, *p* < 0.001], sad [*t*(852) = 11.35, *p* < 0.001], happy [*t*(852) = 6.77, *p* < 0.001], and bored [*t*(852) = 8.4, *p* < 0.001] emotional expressions. No age-effect was found for recognition of interest [*t*(852) = 0.44, *p* = 0.651] (see Figure 3C).

Descriptive data for both age groups can be found in Supplementary Material (see Section S2). It is important to note that recognition performance was neither at the floor nor ceiling (chance level = 0.25).

#### 3.1.3. Age-Related Differences in Eye-Gaze

The proportion of mouth looking data collected in Experiment 3 was analyzed using a 2 × 6 rm ANOVA with Age Group (2; younger adults [YAs], older adults [OAs]) as the between-group variable and Emotion (6; Angry, Friendly, Sad, Happy, Bored, Interested) as the within-group variable. The main effect of Age Group was not significant [F(1, 32)= 2.02, *p* = 0.17, η^2^ = 0.002]. The main effect of Emotion was significant [F(5, 160) = 11.25, *p* < 0.001, η^2^ = 0.54]. Pairwise comparisons revealed significant, more proportional mouth looking for happy compared to all three negative emotions (angry: *p* = 0.01; sad: *p* < 0.001; bored: *p* = 0.001) and friendly (*p* = 0.02). Pairwise comparisons also revealed more proportional mouth looking for friendly (*p* = 0.01) and interested (*p* < 0.001) compared to sad and more proportional mouth looking for angry compared to sad (*p* = 0.02). The interaction between Age Group and Emotion was not significant [F(5, 160)= 0.64, *p* = 0.67, η^2^ = 0.002] (see Figure 3D).

### 3.2. The Positivity Effect

#### 3.2.1. The Positivity Effect in Valence and Arousal

**Figure 4:**
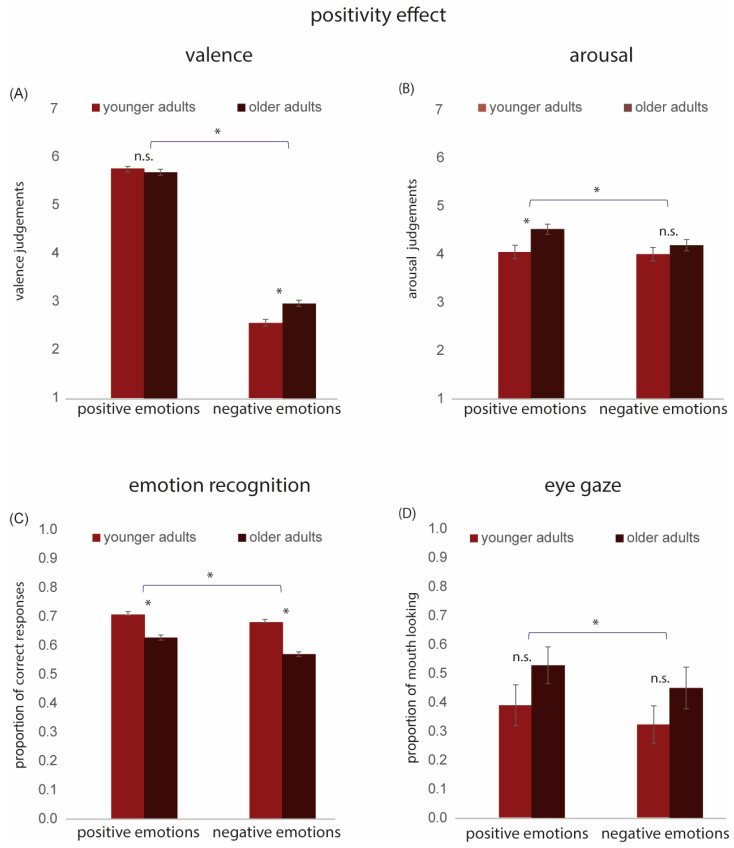
Positivity effect: Results for positive and negative emotions in younger and older adults. (**A**) Mean valence scores. (**B**) Mean arousal scores. (**C**) Mean accuracy of emotion recognition. (**D**) Proportion of mouth looking. Note that high values (>0.5) indicate more looking at the mouth, while lower values (<0.5) suggest more looking at the eyes. * denotes statistically significant effects; n.s. = not significant. Error bars indicate ± 1 standard error.

The valence and arousal ratings collected in Experiment 1 for negative emotions (angry, bored, and sad) and positive emotions (friendly, interested, and happy) were averaged separately for each participant, collapsing across face age. These data were analyzed using a 2 × 2 rm ANOVA with Age Group (2; Young Adults [YAs] and Older Adults [OAs]) as the between-group variable and Emotion Valence (2; Positive Emotions and Negative Emotions) as the within-group variable (see Figure 4A,B).

For valence, the main effects of Age Group [F(1, 127) = 10.96, *p* < 0.001, η^2^ = 0.07] and Emotion Valence [F(1, 127) = 1233.53, *p* < 0.001, η^2^ = 0.90] were significant. The interaction between Age Group and Emotion Valence was also significant [F(1, 127) = 8.16, *p* < 0.01, η^2^ = 0.06]. Post-hoc *t*-tests revealed that OAs rated negative facial expressions as less negative than YAs [*t*(127) = −3.88, *p* < 0.001]. In contrast, there were no significant differences in valence ratings between YAs and OAs for positive emotions [*t*(127) = 0.61, *p* = 0.53].

For arousal, the main effects of Age Group [F(1, 127) = 7.63, *p* < 0.01, η^2^ = 0.05] and Emotion Valence [F(1, 127) = 6.23, *p* < 0.05, η^2^ = 0.04] were significant. The interaction between Age Group and Valence was also significant [F(1, 127) = 6.23, *p* < 0.05, η^2^ = 0.03]. Post-hoc *t*-tests revealed that OAs rated positive emotions as more arousing than YAs [*t*(127) = −3.30, *p* < 0.01]. In contrast, there was no significant difference in arousal ratings between YAs and OAs for negative emotions [*t*(127) = −1.30, *p* = 0.19] (see Figure 4B).

#### 3.2.2. Positivity Effect in Emotion Recognition

We calculated the average proportion of correct responses collected in Experiment 2 for negative emotions (angry, bored, and sad) and positive emotions (friendly, interested, and happy) for each participant. These data were analyzed using a 2 × 2 rm ANOVA with Age Group (younger adults [YAs], older adults [OAs]) as the between-group variable and Emotion Valence (positive, negative) as the within-group variable. The main effects of Age Group [F(1, 852) = 128.61, *p* < 0.001, η^2^ = 0.13 and Emotion Valence [F(1, 852) = 60.61, *p* < 0.001, η^2^ = 0.06] were significant. The interaction between Age Group and Emotion Valence was also significant [F(1, 852) = 8.86, *p* < 0.01, η^2^ = 0.01]; however, the effect size was extremely small. Post-hoc *t*-tests revealed a significantly lower accuracy in emotion recognition for OAs compared to YAs for both positive [*t*(852) = 7.66, *p* < 0.001] and negative [*t*(852) = 12.06, *p* < 0.001] emotions, with the effect of age being slightly stronger for negative emotions (see Figure 4C).

#### 3.2.3. Positivity Effect in Eye-Gaze

The proportion of mouth looking data collected in Experiment 3 was analyzed using a 2 × 2 rm ANOVA with Age Group (2; younger adults [YAs], older adults [OAs]) as the between-group variable and Emotion Valence (2; positive, negative) as the within-group variable. The main effect of Age Group was not significant [F(1, 32) = 1.99, *p* = 0.17, η^2^ = 0.002]. The main effect of Emotion Valence was significant [F(1,32) = 19.86, *p* < 0.001, η^2^ = 0.39]. Pairwise comparisons indicated significantly greater proportional mouth looking for positive compared to negative emotions (*p* = 0.001) (see Figure 4D). The Age Group and Emotion Valence interaction was not significant [F(1, 32) = 0.11, *p* = 0.74, η^2^ = 0.003].

### 3.3. Own-Age Bias

#### 3.3.1. Own-Age Bias in Valence and Arousal

**Figure 5:**
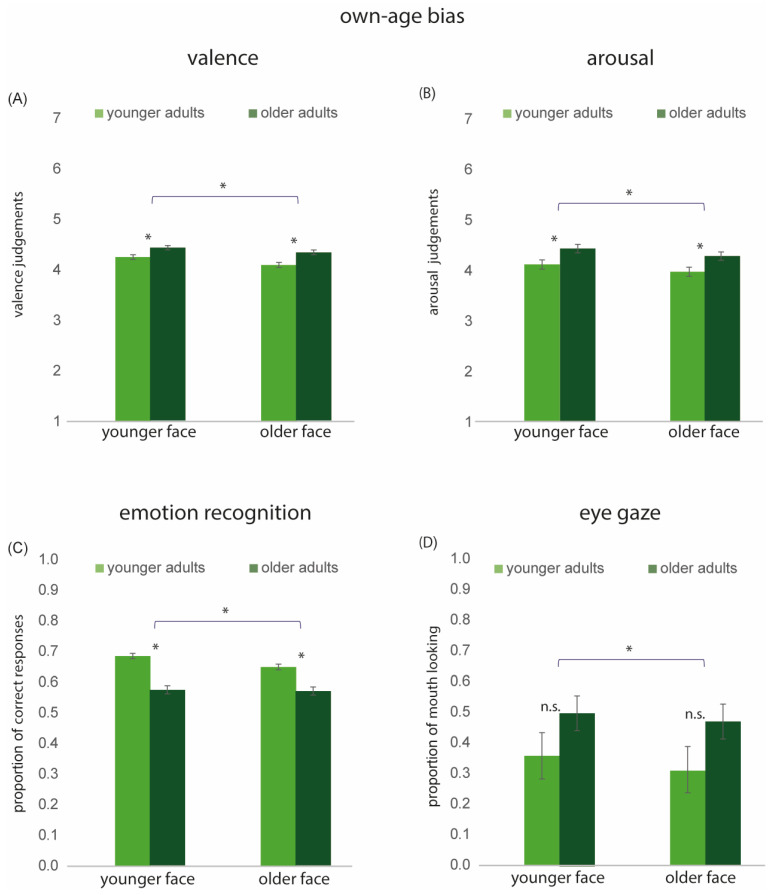
Own-Age bias: Results for younger and older faces in younger and older adults. (**A**) Mean valence scores. (**B**) Mean arousal scores. (**C**) Mean accuracy of emotion recognition. (**D**) Proportion of mouth looking. * denotes statistically significant effects; n.s. = not significant. Error bars indicate ± 1 standard error.

Valence and arousal ratings collected in Experiment 1 were averaged separately for younger faces and older faces for each participant, collapsing across emotions. A 2 × 2 rm ANOVA was conducted with Face Age (2; younger models vs. older models) as the within-group variable and Age Group (2; Younger Adults [YAs] vs. Older Adults [OAs]) as the between-group variable. For valence, the main effects of Age Group [F(1, 127) = 10.96, *p* < 0.01, η^2^ = 0.07] and Face Age [F(1, 127) = 12.76, *p* < 0.001, η^2^ = 0.09] were significant. Both age groups judged emotional expressions presented by younger models as more positive compared to those shown by older models (see Figure 5A). The interaction between Age Group and Face Age was not significant [F(1, 127) = 0.83, *p* = 0.34], η^2^ = 0.006.

For arousal, the main effects of Age Group [F(1, 127) = 7.36, *p* < 0.01, η^2^ = 0.05] and Face Age [F(1, 127) = 12.24, *p* < 0.001, η^2^ = 0.08] were significant. Expressions posed by younger faces were judged as more arousing than those posed by older faces. The interaction between Age Group and Face Age was not significant [F(1, 127) = 0.002, *p* = 0.96, η^2^ = 0.00001], suggesting that the processes underlying the Face Age effect are likely shared across both age groups (see Figure 5B).

#### 3.3.2. Own-Age Bias in Emotion Recognition

Proportions of correct responses collected in Experiment 2 were calculated for each participant across all emotions for both younger and older models. This data was analyzed using a 2 × 2 rm ANOVA with Face Age (2; young model vs. older model) as within-group variable and Age Group (2; Younger Adults [YAs] vs. Older Adults [OAs]) as a between-group variable. The main effects of Age Group [F(1, 852) = 128.61, *p* < 0.001, η^2^ = 0.13] and Face Age [F(1, 852) = 29.74, *p* < 0.001, η^2^ = 0.03] were significant. The interaction between Age Group and Face Age was also significant [F(1, 852) = 19.75, *p* < 0.001, η^2^ = 0.02]. Overall, younger adults outperformed older adults in emotion recognition and the performance was higher when emotions were presented by younger models compared to older models. The interaction indicated that older adults demonstrated equally accurate emotion recognition for younger and older models [*t*(286) = 0.35, *p* = 0.72]. In contrast, younger adults exhibited an own-age bias, showing greater accuracy in recognizing emotions displayed by younger models [*t*(568) = 7.66, *p* < 0.001]) (see Figure 5C).

#### 3.3.3. Own-Age Bias in Eye-Gaze

The proportion of mouth looking collected in Experiment 3 was calculated for each participant across all emotions for both younger and older faces. This data was analyzed using a 2 × 2 rm ANOVA with Age Group (2; younger adults [YAs], older adults [OAs]) as the between-group variable and Face Age (2; younger model vs. older model) as the within-group variable. The main effect of Age Group was not significant [F(1, 32) = 2.05, *p* < 0.16, η^2^ = 0.002]. The main effect of Face Age was significant [F(1, 32) = 7.74, *p* < 0.01; η^2^ = 0.19]. Pairwise comparisons revealed significantly more proportional mouth looking for younger than for older faces (*p* < 0.01). The interaction between Age Group and Face Age was not significant [F(1, 32) = 0.02, *p* < 0.89, η^2^ = 0.0006].

## 4. Discussion

Age-related differences in emotion recognition are well-documented. In this study, we examined how stimulus valence and face age influence this phenomenon across multiple facets of emotion processing. Younger and older participants were shown images from a new stimulus set that portrays younger and older models expressing three positive and three negative emotions. Emotion processing was assessed via valence and arousal ratings (Experiment 1), emotion recognition accuracy (Experiment 2), and eye-tracking patterns (Experiment 3). Our analyses focused on the following questions: (1) How does aging influence the processing of emotional expressions?; (2) How does emotional valence (positive vs. negative) affect emotion processing in young and older adulthood when there is a balance between positive and negative stimuli?; and (3) Does face age (younger vs. older models) influence emotion processing? In this section, we discuss our findings related to each of these research questions, followed by a review of this study’s limitations and a conclusion.

### 4.1. How Does Aging Influence the Processing of Emotional Expressions?

We found significant age-related effects for various aspects of emotion processing, which supports evidence that younger and older adults process emotional expressions differently (Hayes et al., 2020). First, older adults rated all stimuli as more positive in valence and more arousing than younger adults. Second, older adults displayed poorer recognition accuracy overall compared to younger adults. Hence, healthy aging does change the way in which emotional expressions are processed. Several explanations have been proposed to explain these differences including an age-related shift in cognitive resources allocated to positive stimuli (Carstensen et al., 1999), differences in scanning patterns (Campbell et al., 2015; Grainger & Henry, 2020), and changes in brain volume and/or neurotransmitters (Ruffman & Sutcliffe, 2020). Our methods do not allow us to directly examine changes in brain functioning and we refer the reader to Ruffman and Sutcliffe (Ruffman & Sutcliffe, 2020) for more details. With regard to visual scanning patterns, both younger and older participants displayed relatively balanced looking patterns, with a tendency for more fixations on the eyes. There was a tendency for older adults to spend more time looking at the mouth compared to younger adults; however, this effect was not statistically significant (see Figure 3D). In the literature, some studies show a higher proportion of fixations on the mouth for older adults (Firestone et al., 2007; Grainger & Henry, 2020; Sullivan et al., 2007), while others do not (Chaby et al., 2017; Eisenbarth & Alpers, 2011; Firestone et al., 2007; Stam et al., 2022). Given these inconsistencies, and the fact that age-related effects were not significant in the present study, we conclude that differences in eye movements are unlikely to account for age-related differences in emotion processing. Instead, our results suggest a differential treatment of negative vs. positive stimuli, as discussed in greater detail below (see Section 4.2).

### 4.2. How Does Emotional Valence (Positive vs. Negative) Affect Emotion Processing in Younger and Older Adulthood?

In the Introduction, we outlined limitations associated with previous studies that utilized stimulus sets that portray only one positive and many negative emotions. Specifically, the imbalance of one positive vs. many negative emotions makes recognition of the positive emotion easier and ceiling effects are often observed (Hayes et al., 2020; Ruffman et al., 2008). This confounds interpretation of the positivity effect. To address this issue, we utilized a stimulus set where the number of positive and negative emotions is balanced. We found evidence that younger and older adults process positive and negative stimuli differently; however, the pattern we observed suggests a more nuanced picture of the positivity effect than has previously been thought. For emotion recognition accuracy, we found a statistically significant interaction between age group and stimulus valence. This interaction was characterized by a more pronounced age effect for negative emotions. These results are consistent with Cortes et al. (2021) in suggesting that the positivity effect in emotion recognition is greatly reduced or even absent when the number of positive and negative options is balanced. However, this finding must be treated with caution because the effect was very small, and small effects are more likely to be significant when the sample is large.

Both younger and older adults spent more time looking at the lower part (i.e., mouth) of faces posing positive compared to negative expressions. However, this effect did not interact with age group. Moreover, we did not find clear evidence that older adults spend more time looking at the mouth, as discussed in Section 4.1. above. As a whole, these findings are inconsistent with the notion that older adults are better at recognizing positive expressions because they are signaled by the mouth region.

Hence, the results of Experiment 2 and Experiment 3 suggest that the positivity effect does not consistently manifest through accuracy of emotion recognition nor scanning patterns. However, we did find evidence that older adults treat positive emotions differently to negative emotions. Older adults rated negative facial expressions as less negative than younger adults. Moreover, older adults rated positive emotions as being more arousing than younger adults. From the lens of SST, these findings may reflect a motivation to pay more attention to elements that promote positive affect (Carstensen et al., 1999; Charles & Carstensen, 2010; Labouvie-Vief et al., 2010). Similarly, the dynamic integration theory (DIT: Labouvie-Vief, 2009) posits that older adults optimize personal growth and maintain emotional wellbeing by diverting attention away from negative information. Both theories are supported by evidence that older adults prioritize positive over negative information in attention, memory, and the interpretation of ambiguous stimuli (Clark-Foos & Marsh, 2008; Grühn & Scheibe, 2008; Kehoe et al., 2013). Our findings for valence and arousal align with these findings. Our results also suggest that valence and arousal ratings may be more sensitive to age-related shifts in the processing of positive stimuli than other aspects of emotion processing. We can only speculate on the possible reasons for this. One possibility is that age-related differences in emotion recognition reflect changes in multiple underlying functions, including those beyond emotion processing, such as verbal abilities, sensory perception, and personality (Hildebrandt et al., 2015; Mill et al., 2009; Newen et al., 2015). This could also help explain why studies on emotion recognition have produced inconsistent results regarding aging and the positivity effect. Our findings highlight the importance of expanding this area of research beyond the discrete emotion model at both theoretical and methodological levels. For instance, by incorporating complex emotions along with the valence and arousal dimensions, we uncovered manifestations of the positivity effect within the valence–arousal framework.

### 4.3. Does Face Age (Younger vs. Older Models) Influence Emotion Processing?

There is evidence to suggest that an own-age bias occurs in the recognition of facial identity, whereby younger adults are better at recognizing younger faces as compared to older faces (reviewed by Rhodes & Anastasi, 2012). In contrast, older adults perform with similar accuracy for younger and older faces. These effects have been attributed to in-group out-group categorization effects (Rhodes & Anastasi, 2012), which could also have implications for the processing of facial expressions of emotions (Hess et al., 2005). However, the few studies that have examined this question have produced inconsistent results (Ebner et al., 2011; Ebner & Johnson, 2009). Our study aimed to further explore this question. Overall, our results do not support the notion of an own-age bias in emotion processing. Face age influenced the dependent variables measured but the effect of face age did not interact with age group. First, expressions portrayed by younger faces were judged as more intense than those made by older faces. These effects were observed in both age groups, suggesting that the underlying processes are not age dependent. Similarly, both age groups rated expressions portrayed by younger models as more positive compared to expressions portrayed by older models. Finally, participants spent more time looking at the mouth of younger faces compared to older faces. The only evidence for an own-age bias was for emotion recognition accuracy, with younger adults exhibiting a small advantage in recognizing emotions expressed by younger models compared to older models.

It is possible that age-related changes in facial appearance impact emotion processing in several ways: facial cues may elicit ageist stereotypes, reduce cognitive resources allocated to out-group members, and increase difficulty in reading emotional signals due to wrinkles and changes in facial musculature. The appearance of wrinkles and changes in facial musculature can diminish specific emotional cues and/or create the impression of a permanent affective state that accentuates or masks other emotions (Fölster et al., 2014; Grondhuis et al., 2021). These age-related changes in the physical expression of emotions may explain why emotions expressed by younger faces were rated as more intense compared to those portrayed by older adults. It is well documented that attitudes towards older adults are generally negative (North & Fiske, 2012). Moreover, facial cues to aging play a significant role in eliciting these negative attitudes (Hummert et al., 1997). Our finding that younger models were rated more positively aligns with this research. Our findings highlight the intricate relationship between aging and emotion processing and underscore the importance of considering both participant age and face age to achieve a comprehensive lifespan perspective. What makes the study of face age unique is its dynamic nature, tied to the fact that everyone will eventually age. Unlike more static characteristics like race or gender, the relationship individuals have with age changes over time. Because emotion processing plays a key role in successful social interactions, the effects of face age reported here and elsewhere (Ebner et al., 2011; Ebner & Johnson, 2009; Rhodes & Anastasi, 2012) are likely to have a significant impact on intergenerational interactions. Further research is essential to fill gaps related to the limited availability of stimulus sets featuring older models and the small number of studies that incorporate face age as a variable. Such efforts could help unravel this multifaceted phenomenon and provide greater clarity on the heterogeneous findings that have been documented in the literature.

### 4.4. Limitations

One limitation of the present study is that all of the models were women, and the majority of participants were also women. This could be problematic because women may be more adept at emotion recognition; however, these effects are not consistently observed across all emotions and modalities (Alaerts et al., 2011; Connolly et al., 2019; Lambrecht et al., 2014). None of the effects of participant gender were significant in our study (gender effect on emotion recognition: F(1, 848) = 0.54, *p* = 0.46., for Valence: F(1, 125) = 0.16, *p* = 0.68, Arousal: F(1, 125) = 0.25, *p* = 0.61) nor were interactions including gender. Nonetheless, interesting interactions between ageism and gender have been reported in the literature. For example, gray hair is often perceived negatively for older women but is considered distinguished for older men (Rochon et al., 2021). It will be important for future research to examine the intersection of age-related and gender-related biases in emotion processing.

The sample size for Experiment 3 was small, which may explain the higher variability observed for the eye tracking data compared to the behavioral data. This complicates the interpretation of non-significant results, particularly when comparing the two age groups. The challenges associated with obtaining high-quality eye-tracking data from older adults likely account for the small sample sizes commonly seen in this area of research (Dalrymple et al., 2018). Additional studies are needed, and with sufficient data, a meta-analysis could eventually clarify existing inconsistencies.

It has been argued that static expressions do not reflect the dynamic nature of emotion communication (Isaacowitz & Stanley, 2011). We focused on static displays in the present study for pragmatic reasons. First, most research on aging and emotion processing has relied on static displays. Using static displays here facilitates comparison with this extant literature. There are also challenges associated with the acquisition of dynamic displays, especially when one wishes to capture more natural emotions expressed by non-actors. Finally, age-related changes in the speed of processing may accentuate impairments in the processing of dynamic emotions in older adults and complicate the interpretation of age-related effects (Hayes et al., 2020). It is important to note that Cortes et al. (Cortes et al., 2021), who utilized dynamic displays in their study, also reported a diminished positivity effect with a balanced number of positive and negative emotions. It is therefore unlikely that the use of static displays in our study drove this effect. Nonetheless, it would be valuable for future research to investigate whether interpretations of dynamic displays yield similar results to those reported here for the valence and arousal dimensions.

## 5. Conclusions

Our goal was to investigate how aging influences emotion processing from both the observers’ (i.e., participant) and expressers’ (i.e., face model) perspectives. Additionally, we extended previous research by examining the positivity effect with a broader range of positive emotions and by incorporating various aspects of emotion processing, including valence–arousal dimensions, recognition accuracy, and eye-tracking measures. Adopting this comprehensive approach allowed us to uncover nuances in emotion processing across the lifespan of any adult. Older adults had more difficulty than younger adults in recognizing emotions in our new stimulus set. Age-related differences in scanning patterns were not statistically significant and overall, both age groups displayed comparable exploratory strategies. Despite the near-absence of a positivity effect in emotion recognition in our study (i.e., a statistically significant effect with a small effect size), we did find evidence that older adults treat positive emotions differently to negative emotions for the valence–arousal dimensions. Finally, younger adult faces were rated as more intense and more positive than older adult faces. We conclude that participant age, stimulus valence, and model age shape emotion processing in both overlapping and distinct ways across various manifestations of this complex phenomenon. Beyond their implications for theories of human aging, studies on emotion processing have real-world significance. Indeed, the age-related and stimulus valence effects observed here and in other studies likely influence intergenerational interactions. Future research should build on this work by integrating diverse measures of emotion processing into their study designs.

## Figures and Tables

**Table 1:** Sample demographic characteristics in Experiments 1–3 (Exp. 1–3). Edu (=Education) shows the percentage of the participants with higher education (Bachelor’s degree and higher) in each age group (YAs = young adults; OAs = older adults).

	Group	N	Mean Age	Min. Age	Max. Age	Gender			Edu High
						F	M	D	
Exp. 1	YAs	59	28	18	35	37	22	0	40%
	OAs	70	68	60	79	44	26	0	40%
Exp. 2	YAs	568	24.3	18	39	439	127	2	16%
	OAs	286	68.4	60	87	171	115	0	18%
Exp. 3	YAs	17	24.2	18	34	11	6	0	23%
	OAs	17	68.7	60	75	12	5	0	21%

## Data Availability

All data are fully available on the OSF platform: https://doi.org/10.17605/OSF.IO/8K3X5.

## Supplementary Materials

The following supporting information can be downloaded at: https://www.mdpi.com/article/10.3390/bs15030302/s1, Figure S1: Distractor construction procedure; Figure S2: Mean proportion of correct responses (pc) in three waves of data collection for Experiment 2. Table S1: List of pictures, target emotions and distractors in German language; Table S2: List of pictures, target emotions and distractors in English language; Table S3: Descriptive statistics; Table S4: Reliability.
