# Supplementary material for: Emotion Processing in Late Adulthood: The Effect of Emotional Valence and Face Age on Behavior and Scanning Patterns
Source: Behav Sci (Basel). 2025 Mar 4;15(3):302. doi: 10.3390/bs15030302 (PMC11939290; doi:10.3390/bs15030302)
Supplement: Supplementary file 1 [file behavsci-15-00302-s001.zip › behavsci-3379963-supplementary.pdf]

Supplementary Material

Section S1: Development of the Facial Expression in Aging Test (FEAT)

Picture selection procedure: The two best pictures of each emotional state of a person were pre-selected by authors. This resulted in a set of 120 images, showing 20 models and 6 different emotional states. Afterwards, a group of 20 younger participants (psychology students) were asked to rate emotion intensity of the target emotion (i.e., angry) in preselected images (not et al angry– very angry - see Fig 1S/A). Based on the ratings, we selected pictures with the highest ratings (all > 3.0 on target emotions). As result, a final set of 72 pictures was selected. Four models were excluded from the final Facial Expression in Aging Test (FEAT) because of low facial expressivity and low specificity of emotions.

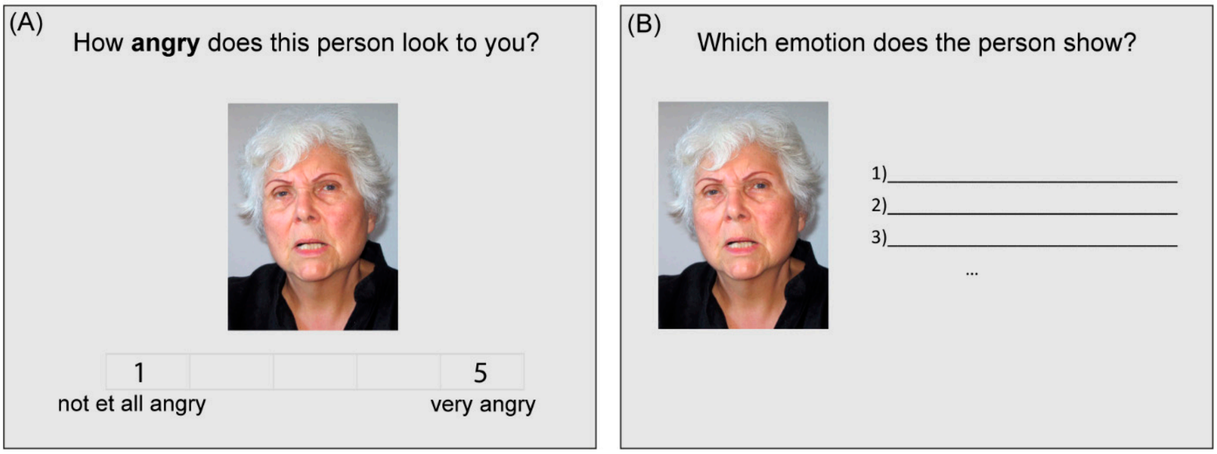

Figure S1: Distractor construction procedure A) An example of the rating procedure that was used to select the final set of images for the FEAT B) An example of the item used to generate distractor words: Participants were allowed to name as many emotions as they wished.

Distractor construction procedure: In order to construct ecologically valid distractors for each target emotion and each stimulus picture, we recruited a new sample of 20 psychology students. Participants were asked to write down all emotions they could recognize in each of the 72 emotional pictures (see Fig. 1S/B).

In total, for all images showing an *angry* face, 190 responses were given, with about 50% of words being classified as synonyms for angry (i.e., annoyed, irritated, aggressive, stunned). Total of emotional word generated for pictures showing a *bored* emotional expression was 163, but again there were many synonyms (i.e., disinterested, inattentive, dull). For target emotion *sad* there were in total 120 responses including synonyms (i.e., despondent, gloomy, dejected). Some emotional states were found frequently for different negative target emotions (i.e., disappointed was frequently reported for angry, sad and bored faces). For *friendly* faces we had 95 responses in total (including synonyms such as pleasant, kind, affable). For faces showing *interest* there were in total 142 responses, including synonyms (i.e., attentive, engaged, focused), and for happy faces there were 129 responses, including synonyms (i.e. cheerful, enjoyable). Somewhat surprisingly, for positive emotional pictures there were approximately 15% responses with negative valance (i.e., disappointed, serious, bored, resigned; mischievous, irritated).

We used the frequency of the distractor words to select appropriate distractors. Only those distractor words that had not been repeatedly reported (and thus were not consistently misinterpreted as the target emotion) were selected. Additionally, we included distracting words from other negative emotions. For example, the distractors for anger were created using words associated with emotions "bored" and "sad." Furthermore, the distractors were created for each picture individually, as we were able to track which distractors were reported for each face model. Based on responses for each individual image and distractors generated for the set of all target emotions, we created a final list of distractors for each of the 72 stimuli images. The list of distractors in German is provided in Table 1S. The emotion labels have been also translated in English (see Table S2). Moreover, as recommended for complex images, we calculated RMS contrast for each image, defined as the standard deviation of normalized grey values, ˆu = u/255, 0 ≤ ˆu i ≤ 1 (Peli, 1990). No significant differences in contrast were observed for images depicting different Emotions [$F(5, 60)=.68, p=.63, \eta 2=.05$], nor for images of Younger versus Older models [$F(1, 60)=.86, p=.36, \eta 2=.01$]. Additionally, the interaction between Emotion and Age of model was not significant [$F(5, 60)=.95, p=.45, \eta 2=.07$].

Table S1: List of pictures, target emotions and distractors in German language. Note that the image name carries a code about the model, age, and emotion. For example: 4Afriendly = model 4; older face, friendly emotional expression or 6Jsad.tif = model 6; younger face, sad emotional expression. RMS Cont = Contrast.

Image Name	RMS Cont	Target Emotion	Distractor	Distractor	Distractor
	**freundlich**				
4Afriendly.tif	0.26	freundlich	sorgenlos	aufmerksam	ängstlich
20Jfriendly.tif	0.19	freundlich	niedergeschlagen	sorgenlos	schadenfroh
5Afriendly.tif	0.22	freundlich	begeistert	spielerisch	zweifelnd
13Jfriendly.tif	0.13	freundlich	interessiert	erstaunt	schadenfroh
6Jfriendly.tif	0.22	freundlich	stolz	herausfordernd	erfüllt
10Afriendly.tif	0.14	freundlich	skeptisch	neugierig	spielerisch
2Afriendly.tif	0.18	freundlich	neugierig	erwartungsvoll	aufgeweckt
19Jfriendly.tif	0.27	freundlich	nachdenklich	zärtlich	neugierig
18Afriendly.tif	0.28	freundlich	aufgeschlossen	hoffnungsvoll	sorgenlos
14Jfriendly.tif	0.24	freundlich	verspielt	überrascht	neugierig
1Jfriendly.tif	0.28	freundlich	unsicher	verträumt	begeistert
17Afriendly.tif	0.21	freundlich	interessiert	unentschlossen	verzweifelt
	**fröhlich**				
18Ajoyful.tif	0.29	fröhlich	aufgeweckt	höflich	unentschlossen
14Jjoyful.tif	0.23	fröhlich	herausfordernd	zugetan	aufgeschlossen
6Jjoyful.tif	0.22	fröhlich	überrascht	interessiert	abgelenkt
17Ajoyful.tif	0.23	fröhlich	unsicher	nachdenklich	zuvorkommend
16Ajoyful.tif	0.29	fröhlich	aufmerksam	interessiert	erwartungsvoll
8Jjoyful.tif	0.18	fröhlich	verspielt	zärtlich	höflich
11Ajoyful.tif	0.26	fröhlich	neugierig	ängstlich	verträumt
13Jjoyful.tif	0.19	fröhlich	neugierig	zweifelnd	schadenfroh
2Ajoyful.tif	0.17	fröhlich	neugierig	verträumt	höflich
19joyful.tif	0.23	fröhlich	neugierig	offen	skeptisch
7Ajoyful.tif	0.20	fröhlich	entspannt	stolz	zuvorkommend

20Jjoyful.tif	0.16	fröhlich	spielerisch	zugetan	verzweifelt
	**gelangweilt**				
2Abored.tif	0.20	gelangweilt	ernst	verärgert	trotzig
19Jbored.tif	0.23	gelangweilt	erwartungsvoll	abgelenkt	verärgert
6Jbored.tif	0.22	gelangweilt	verärgert	irritiert	erstaunt
17Abored.tif	0.25	gelangweilt	traurig	verlegen	entsetzt
7Abored.tif	0.18	gelangweilt	unsicher	nachdenklich	traurig
20Jbored.tif	0.21	gelangweilt	zweifelnd	erwartungsvoll	verärgert
1Jbored.tif	0.27	gelangweilt	traurig	nachdenklich	entsetzt
10Abored.tif	0.16	gelangweilt	bestimmend	misstrauisch	traurig
11Abored.tif	0.23	gelangweilt	traurig	angewidert	zurückhaltend
13Jbored.ai	0.25	gelangweilt	traurig	zurückhaltend	resigniert
18Abored.tif	0.27	gelangweilt	verwirrt	arrogant	verärgert
14Jbored.tif	0.24	gelangweilt	unentschlossen	verärgert	angewidert
	**interessiert**				
8Jinterested.tif	0.32	interessiert	erstaunt	skeptisch	unsicher
9Ainterested.tif	0.29	interessiert	fröhlich	stolz	verzweifelt
1Jinterested.tif	0.28	interessiert	fröhlich	unentschlossen	abgelenkt
4Ainterested.tif	0.27	interessiert	abgelenkt	freundlich	offen
13Jinterested.tif	0.23	interessiert	erstaunt	höflich	verzweifelt
18Ainterested.tif	0.29	interessiert	verzweifelt	verspielt	fröhlich
10Ainterested.tif	0.14	interessiert	ängstlich	spielerisch	freundlich
20Jinterested.tif	0.21	interessiert	niedergeschlagen	höflich	skeptisch
7Ainterested.tif	0.20	interessiert	höflich	nachdenklich	zweifelnd
19Jinterested.tif	0.25	interessiert	überrascht	fröhlich	verzweifelt
6Jinterested.tif	0.22	interessiert	fröhlich	aufgeschlossen	entspannt
16Ainterested.tif	0.24	interessiert	skeptisch	freundlich	zärtlich
	**traurig**				
6Jsad.tif	0.24	traurig	verärgert	abgelenkt	bestimmend
2Asad.tif	0.17	traurig	verwirrt	gelangweilt	irritiert
14Jsad.tif	0.26	traurig	entsetzt	verärgert	resigniert
18Asad.tif	0.26	traurig	verärgert	trotzig	unentschlossen
3Jsad.tif	0.30	traurig	verärgert	misstrauisch	angespannt
4Asad.tif	0.23	traurig	skeptisch	gelangweilt	verlegen
7Asad2.tif	0.20	traurig	zweifelnd	erwartungsvoll	gelangweilt
8Jsad.tif	0.18	traurig	missbilligend	ablehnend	verärgert
1Jsad.tif	0.25	traurig	ernst	nachdenklich	verärgert
9Asad.tif	0.25	traurig	zurückhaltend	unsicher	gelangweilt
20Jsad.tif	0.22	traurig	abwesend	verärgert	erschrocken
7Asad1.tif	0.16	traurig	arrogant	verärgert	irritiert
	**verärgert**				
2Aangry.tif	0.20	verärgert	nachdenklich	verwirrt	traurig
1Jangry1.tif	0.23	verärgert	ablehnend	irritiert	traurig
7Aangry.tif	0.19	verärgert	zurückhaltend	erschrocken	gelangweilt
20Jangry.tif	0.21	verärgert	gelangweilt	bestimmend	trotzig

8Jangry.tif	0.25	verärgert	traurig	skeptisch	zweifelnd
11Aangry.tif	0.28	verärgert	erstaunt	angewidert	traurig
3Jangry.tif	0.29	verärgert	unsicher	gelangweilt	arrogant
16Aangry.tif	0.25	verärgert	traurig	ernst	erwartungsvoll
10Aangry.tif	0.17	verärgert	erstaunt	gelangweilt	ablehnend
13Jangry.tif	0.21	verärgert	ratlos	gelangweilt	unentschlossen
17AangryI.tif	0.24	verärgert	gelangweilt	zurückhaltend	erwartungsvoll
19Jangry.tif	0.26	verärgert	misstrauisch	angespannt	traurig

Table S2: List of pictures, target emotions and distractors in English language.

Image Name	RMS Cont	Target Emotion	Distractor	Distractor	Distractor
	**kind**				
4Afriendly.tif	0.26	friendly	carefree	attentive	anxious
20Jfriendly.tif	0.19	friendly	dejected	carefree	malicious
5Afriendly.tif	0.22	friendly	thrilled	playful	doubtful
13Jfriendly.tif	0.13	friendly	interested	astonished	malicious
6Jfriendly.tif	0.22	friendly	proud	provocative	fulfilled
10Afriendly.tif	0.14	friendly	skeptical	curious	playful
2Afriendly.tif	0.18	friendly	curious	expectant	lively
19Jfriendly.tif	0.27	friendly	pensive	gentle	curious
18Afriendly.tif	0.28	friendly	open-hearted	hopeful	carefree
14Jfriendly.tif	0.24	friendly	frisky	surprised	curious
1Jfriendly.tif	0.28	friendly	uncertain	dazed	thrilled
17Afriendly.tif	0.21	friendly	interested	indecisive	desperrate
	**happy**				
18Ajoyful.tif	0.29	happy	lively	polite	indecisive
14Jjoyful.tif	0.23	happy	provocative	attached	open-hearted
6Jjoyful.tif	0.22	happy	surprised	interested	distracted
17Ajoyful.tif	0.23	happy	uncertain	pensive	accomodating
16Ajoyful.tif	0.29	happy	attentive	interested	expectant
8Jjoyful.tif	0.18	happy	frisky	gentle	polite
11Ajoyful.tif	0.26	happy	curious	anxious	dazed
13Jjoyful.tif	0.19	happy	curious	doubtful	malicious
2Ajoyful.tif	0.17	happy	curious	dazed	polite
19joyful.tif	0.23	happy	curious	open-minded	skeptical
7Ajoyful.tif	0.20	happy	relaxed	proud	accomodating
20Jjoyful.tif	0.16	happy	playful	attached	desperate
	**bored**				
2Abored.tif	0.20	bored	serious	angry	defiant
19Jbored.tif	0.23	bored	expectant	distracted	angry
6Jbored.tif	0.22	bored	angry	puzzled	astonished
17Abored.tif	0.25	bored	sad	embarrassed	appalled

7Abored.tif	0.18	bored	uncertain	pensive	sad
20Jbored.tif	0.21	bored	doubtful	expectant	angry
1Jbored.tif	0.27	bored	sad	pensive	appaled
10Abored.tif	0.16	bored	authoritative	suspicious	sad
11Abored.tif	0.23	bored	sad	disgusted	reluctant
13Jbored.ai	0.25	bored	sad	reluctant	despondent
18Abored.tif	0.27	bored	confused	arrogant	angry
14Jbored.tif	0.24	bored	indecisive	angry	disgusted
	**interested**				
8Jinterested.tif	0.32	interested	astonished	skeptical	uncertain
9Ainterested.tif	0.29	interested	cheerful	proud	desperate
1Jinterested.tif	0.28	interested	cheerful	indecisive	distracted
4Ainterested.tif	0.27	interested	distracted	kind	open-minded
13Jinterested.tif	0.23	interested	astonished	polite	desperate
18Ainterested.tif	0.29	interested	desperate	frisky	cheerful
10Ainterested.tif	0.14	interested	anxious	playful	kind
20Jinterested.tif	0.21	interested	dejected	polite	skeptical
7Ainterested.tif	0.20	interested	polite	pensive	doubtful
19Jinterested.tif	0.25	interested	surprised	cheerful	desperate
6Jinterested.tif	0.22	interested	cheerful	open-hearted	relaxed
16Ainterested.tif	0.24	interested	skeptical	kind	gentle
	**sad**				
6Jsad.tif	0.24	sad	angry	distracted	authoritative
2Asad.tif	0.17	sad	confused	bored	puzzled
14Jsad.tif	0.26	sad	appalled	angry	despondent
18Asad.tif	0.26	sad	angry	defiant	indecisive
3Jsad.tif	0.30	sad	angry	suspicious	on edge
4Asad.tif	0.23	sad	skeptical	bored	embarrassed
7Asad2.tif	0.20	sad	doubtful	expectant	bored
8Jsad.tif	0.18	sad	disapproving	deprecating	angry
1Jsad.tif	0.25	sad	serious	pensive	angry
9Asad.tif	0.25	sad	reluctant	uncertain	bored
20Jsad.tif	0.22	sad	absent	angry	startled
7Asad1.tif	0.16	sad	arrogant	angry	puzzled
	**angry**				
2Aangry.tif	0.20	angry	pensive	confused	sad
1Jangry1.tif	0.23	angry	deprecating	puzzled	sad
7Aangry.tif	0.19	angry	reluctant	startled	bored
20Jangry.tif	0.21	angry	bored	authoritative	defiant
8Jangry.tif	0.25	angry	sad	skeptical	doubtful
11Aangry.tif	0.28	angry	astonished	disgusted	sad
3Jangry.tif	0.29	angry	uncertain	bored	arrogant
16Aangry.tif	0.25	angry	sad	serious	expectant
10Aangry.tif	0.17	angry	astonished	bored	deprecating

13Jangry.tif	0.21	angry	baffled	bored	indecisive
17AangryI.tif	0.24	angry	bored	reluctant	expectant
19Jangry.tif	0.26	angry	suspicious	on edge	sad

Section S2: Descriptive Statistics for the three data collection waves in Experiment 2

Table S3: Descriptive statistics for young and older adults in Wave I, II and III respectively.

		Wave I					Wave II					Wave III				
**Age**	***N***	***Emotion***	***Mean***	***Min***	***Max***	***Std. Dev***	***N***	***Mean***	***Min***	***Max***	***Std. Dev***	***N***	***Mean***	***Min***	***Max***	***Std. Dev***
**YA**	245	angry	.51	.083	.75	.13	188	.64	.25	1.0	.15	135	.67	.33	1.0	.14
		bored	.57	.08	1,0	.18		.62	.08	1.0	.18		.63	.25	1.0	.17
		sad	.73	.25	1.0	.16		.75	.33	1.0	.17		.80	.33	1.0	.15
		friendly	.69	.16	1.0	.18		.73	.16	1.0	.20		.77	.33	1.0	.16
		interested	.50	.083	.83	.14		.64	.16	1.0	.16		.66	.25	1.0	.18
		happy	.72	.16	1.0	.19		.66	.25	1.0	.16		.77	.33	1.0	.15
**OA**	47	angry	.39	.08	.66	.16	96	.54	.16	.83	.14	143	.56	.08	1.0	.16
		bored	.43	.08	.83	.16		.47	.08	.91	.19		.52	.08	1.0	.17
		sad	.56	.25	1.0	.16		.57	.16	.91	.18		.65	.25	1.0	.18
		friendly	.57	.08	.91	.20		.58	.08	1.0	.23		.58	.16	1.0	.18
		interested	.48	.16	.83	.15		.62	.25	.91	.15		.58	.16	1.0	.15
		happy	.60	.25	1.0	.20		.60	.29	.89	.14		.65	.16	1.0	.20

Section S3: Inferential statistics for the three data collection waves in Experiment 2

Prior to pooling data from the three Waves into one data set, we conducted a 3x2x6 rmANOVA with Wave (3; I-III) and Age Group (2; YA, OA) as between-group variables, and Emotion (6; angry, bored, sad, friendly, interested, happy) as within-group variable. We focus here on effects involving the Wave variable. Results involving the other variables are described and discussed in detail in the manuscript. The main effect of Wave was weak but significant ($F(2, 848)=35.61$, $p <. 001$, $\eta 2 =.07$). Bonferroni post-hoc test revealed significant differences between all three waves, such that performance significantly increased from Wave I to Wave III. The main effect of Age Group was significant ($F(1, 848)=190{,}71$, $p < .001$; $\eta 2=18$), but Age Group x Wave interaction was not significant ($F(4, 848)= .55$, $p = .57$; $\eta 2=.001$). Data are shown in Fig. S2.

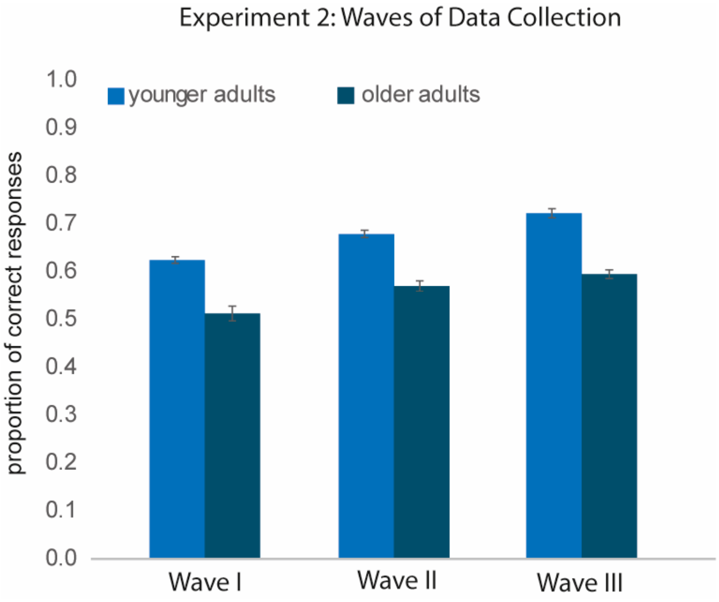

Figure S2: Mean proportion of correct responses (pc) in three waves of data collection for Experiment 2. Although the performance increased, the age effect remained stable over the waves.

Section S4: Reliability

Cronbach's α was overall acceptable, for all three data collection waves in Experiment 2 and both age groups. When calculated for different type of stimuli, the reliability was lower than for the test as whole.

Table S4: Reliability was calculated both separately and in combination for all three waves, as well as for young and older faces.

	**overall**	*Older Faces*	*Younger Faces*	*Negative Emotions*	*Positive Emotions*
**Wave 1**	**.73**	.51	.68	.67	.74
**Wave 2**	**.72**	.61	.55	.63	.80
**Wave 3**	**.76**	.60	.68	.72	.73
**Wave 1-3**	**.77**	.58	.63	.62	.71
**Young adults**	**.75**	.57	.59	.57	.69
**Older adults**	**.71**	.48	.54	.56	.67

While reliability is rarely reported in the literature, our results are comparable to those available when it is reported. We refer the reader to Olderbak et al. (2021) for a review.
